# Emergency department-initiated tobacco control: a randomised controlled trial in an inner city university hospital

**DOI:** 10.1136/tc.2008.028753

**Published:** 2009-06-14

**Authors:** B Neuner, E Weiss-Gerlach, P Miller, P Martus, D Hesse, C Spies

**Affiliations:** 1 Department of Anesthesiology and Intensive Care Medicine, Charité – Universitaetsmedizin Berlin, Campus Virchow-Klinikum and Campus Charité Mitte, Berlin, Germany; 2 Center for Drug and Alcohol Programs, Medical University of South Carolina, Charleston, South Carolina, USA; 3 Institute of Biostatistics and Clinical Epidemiology, Charité – Universitaetsmedizin Berlin, Campus Charité Mitte, Berlin, Germany

## Abstract

**Objectives:**

Emergency department (ED) patients show high smoking rates. The effects of ED-initiated tobacco control (ETC) on 7-day abstinence at 12 months were investigated.

**Methods:**

A randomised controlled intention-to-treat trial (trials registry no.: ISRCTN41527831) was conducted with 1044 patients in an urban ED. ETC consisted of on-site counselling plus up to four telephone booster sessions. Controls received usual care. Analysis was by logistic regression.

**Results:**

In all, 630 (60.7%) participants were males, the median age was 30 years (range 18–81) and the median smoking intensity was 15 (range 1–60) cigarettes per day. Overall, 580 study participants (55.6%) were unmotivated, 331 (31.7%) were ambivalent and 133 (12.7%) were motivated smokers. ETC (median time 30 (range 1–99) min) was administered to 472 (91.7% out of 515) randomised study participants. At follow-up, 685 study participants (65.6% of 1044) could be contacted. In the ETC group, 73 out of 515 (14.2%) in the ETC group were abstinent, whereas 60 out of 529 (11.3%) controls were abstinent (OR adjusted for age and gender = 1.31 (95% CI 0.91 to 1.89, p = 0.15). Stratified for motivation to change behaviour, the adjusted ORs for ETC versus usual care were OR = 1.00 (95% CI 0.57 to 1.76) in unmotivated smokers, respectively OR = 1.37 (95% CI 0.73 to 2.58) in ambivalent smokers and OR = 2.19 (95% CI 0.98 to 4.89) in motivated smokers, p for trend = 0.29.

**Conclusions:**

ETC, in the form of on-site counselling with up to four telephone booster sessions, showed no overall effect on tobacco abstinence after 12 months. A non-significant trend for a better performance of ETC in more motivated smokers was observed.

Emergency department (ED) patients show a prevalence of smoking that exceeds the smoking prevalence in the general population.^1^ Furthermore, ED patients often have limited access to medical care and in particular to health promotion services.^2^ With respect to the role of EDs in delivering preventive services and improving public health,^3^ in October 2006, a joint statement of the American Emergency Medicine Organizations encouraged ED administrators to implement ED-initiated tobacco control (ETC) services and researchers to conduct evaluations of such efforts.^2^ A systematic review from 2002^4^ on the diagnosis and management of smoking and smoking-related illness in the ED identified 2 ED-based studies: a randomised controlled trial (RCT) with 152 study participants found no difference in quit rates (at 3-month follow-up) in those receiving standardised, scripted counselling including referral to a smoking cessation program together with a “Stop Smoking” pamphlet from the American Heart Association compared to controls who only received the pamphlet. None of the intervention group joined the smoking cessation program.^5^ The second study, which was based in a military ED, identified 42 out of 86 smokers who were interested in quitting, of whom 40 were randomised to receive either a formal smoking cessation program or a brief counselling from the ED doctor. None of the study participants completed the smoking cessation programme and only one patient in the brief counselling group had stopped smoking at 6-month follow-up.^6^

Later investigations included a non-controlled feasibility study of health promotion in an ED setting. Of 411 smokers who accepted referral to a smoking cessation programme, 158 were contacted at follow-up. The quit rate was 12%, and another 40% reported reduced smoking.^7^ Another feasibility study with 39 study participants in a tertiary-care ED found no difference in the 7-day abstinence at 6 months in either the intervention (telephone counselling through a tobacco quitline) or control (self-help manual) conditions.^8^ In an RCT with 74 adolescents aged 14 to 19 years old in a university-affiliated hospital ED, no differences in quit rates were found between on-site motivational interviewing plus stage-based take-home material compared with usual care during the 60 month follow-up.^9^ ^10^ Bock *et al*^11^ randomised 543 adult smokers in an observation unit of a university-based ED to either on-site motivational interviewing in combination with 2 telephone booster sessions or a control condition receiving a printed referral sheet with information on local smoking cessation resources. In a per-protocol analysis the odds ratio for a 7-day abstinence at 1, 3 and 6 months was 1.62 (95% confidence interval (CI) 1.05 to 2.50) in the intervention group versus the control group. Boudreaux *et al*^12^ in their pilot study with 90 adult smokers in an urban, academic level 1 trauma centre found a 7-day abstinence at a 3-month follow-up between 6% (standard referral group) and 14% (motivational interviewing per phone in combination with a posted tailored “motivational tool” which contained (among others) a “Personalised Feedback Form” and a “Pro/Con Worksheet”). Thus, due to the relative heterogeneity of the type and duration of ETC tested in previous studies, the superiority of ETC over no treatment conditions remains unclear.

Therefore, the aim of this study was to evaluate the effectiveness of ETC, combining on-site counselling with telephone booster sessions in a large sample of ED patients.

## MATERIALS AND METHODS

### Sample

Study participants were recruited in the ED at the Charité – Universitaetsmedizin Berlin between 06 October 2005 and 21 December 2006. This inner city ED in the very centre of Berlin treats approximately 40 000 patients annually. After ethical committee approval, all patients 18 years of age or older treated in the ED were screened for tobacco use. Those reporting a minimum of one cigarette smoked per day during the last 7 days were asked to participate in the study. Excluded were (1) patients with no capacity to understand the study information and/or no capacity to give informed consent because of acute or chronic mental or physical conditions (intoxication, severe injuries or organic conditions which required immediate medical care, chronic mental illness, blindness, deafness, other handicaps), (2) those with no capacity to operate the computerised screening tool (see below), (3) patients in police custody, (4) homeless patients (or those not reachable for telephone follow-up for other reasons), (5) patients unable to read and understand the informed consent or the screening questions because of language barriers, (6) inpatients treated in the ED, (7) patients treated in the ED not for emergency reasons, as well as (8) staff members of the hospital. Recruiting times were 8 am to 4 pm in week 1, 1 pm to 9 pm in week 2. Additionally, one Saturday per month patients were recruited from 11 am to 9 pm.

### Sample size

At the time of the study enrolment, limited evidence on ETC efficacy (see above) did not allow a calculation of effect sizes. Therefore, the sample size calculation was based on tobacco control findings in hospitalised patients: tobacco control showed superiority over usual care conditions only with a combination of an initial intervention during hospital stay followed by supportive contacts for at least 1 month after discharge.^13^ Assuming a rate of 20% abstinence in the control group^13^ and an effect size of the intervention of 1.8 (95% CI 1.5 to 2.2),^13^ with an α error of 5% and a power of 80%, nQuery Advisor, V. 3.0 (Statistical Solutions, Saugus, Massachusetts, USA) calculated without continuity correction a study size of n1 (intervention) = n2 (controls) = 244 study participants. With an expected loss to follow-up of 50% of smokers over 12 months,^14^ the overall study size was established at 976. We assumed additional losses of 5% (n = 49) due to inappropriate allocation or incomplete baseline screening and thus the target size for study inclusion was n = 1024 randomised participants.

### Randomisation

After written informed consent, all consecutive patients were administered a computerised screening tool. Study participants were randomly assigned to either the ETC group or the control group, stratified for age (three age groups: 18–25 years, 26–40 years and ⩾41 years), gender and motivation to change smoking behaviour (unmotivated, ambivalent and motivated smokers; see below) based on the first three questions of the computerised screening tool. Neither study participants nor staff members on-site knew about the allocation, but the two senior researchers responsible for the ETC (BN, EWG) were informed via short message service (SMS) about a positive allocation. Study participants received this information after they had completed the questionnaire.

### Baseline screening

The computerised screening tool at baseline, using a mouse-only technique requiring no typing, consisted of a question on motivation to change smoking behaviour according to the transtheoretical model of behaviour change:^15^ “When do you wish to stop smoking?”. Those answering “Not within the next 6 months” were considered unmotivated smokers (ie, smokers in the precontemplation stage), those answering “Within the next 6 months but not within the next 4 weeks” were considered ambivalent smokers (ie, smokers in the contemplation stage) and those answering “Within the next 4 weeks” were considered to be motivated smokers (ie, smokers in the preparation stage).^16^ Study participants were additionally administered the validated German version^17^ of the Fagerstroem test for nicotine dependence (FTND),^18^ a detailed smoking history which includes questions on smoking history (duration of smoking, age at onset, attempts to quit during the last 12 months and partner’s smoking status).^19^ Further questions focused on alcohol consumption (hazardous alcohol consumption defined as ⩾5 points on the Alcohol Use Disorder Identification Test – Primary Care (AUDIT-PC)^20^ scale), illicit drug consumption,^14^ and socioeconomic parameters according to the German Health Survey 1998.^21^ Overall, a maximum of 80 questions were asked, which took approximately 20 min. The questioning took place in the ED during waiting times for treatment or diagnostic procedures. Routine medical treatment was given priority over study assessment procedures.

### Intervention

**Figure 1:**
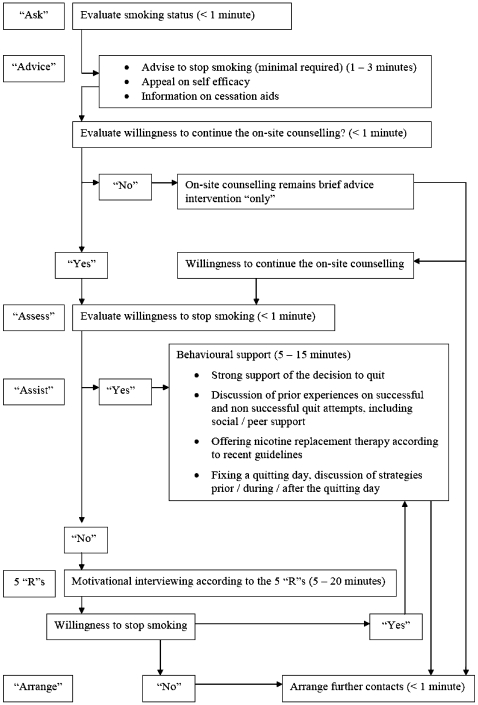
Stages of the on-site counselling in the Emergency Department.

Study participants in the intervention group received ETC in the form of an on-site counselling session and telephone booster follow-up sessions (TBSs). The ETC was conducted according to recent guidelines on tobacco control from the Association of the Scientific Medical Societies in Germany,^22^ based on the meta-analytical findings of Fiore,^23^ Silagy^24^ and West *et al*.^25^ ETC consisted of a smoking cessation intervention using motivational techniques and aimed at unmotivated or ambivalent smokers who were willing to undergo on-site counselling of more than 1–3 min. In motivated smokers, the focus of the intervention was on behavioural support. Overall, the principle of the five “A”s was applied (“ask”, “advice”, “assess”, “assist” and “arrange”)^23^ as shown in fig 1. All patients were given strong advice to quit. In study participants willing to continue on-site counselling but unwilling to agree on a quitting day, motivational interviewing according to the five “R”s (“relevance”, “risks”, “rewards”, “roadblocks” and “repetition”) was performed.^23^ Study participants willing to stop smoking or to reduce their smoking were offered behavioural support^25^ including nicotine replacement therapy (gum, patch, sublingual tablets) according to recent guidelines.^22^ Nicotine replacement therapy was provided free of charge on site. The primary goal of ETC was to motivate participants to quit or to agree on a quit date. The aim of the TBSs was to either further motivate the study participant toward behavioural change (“repeat”/“assist” components) and to “arrange” quit attempts or the maintenance of nicotine abstinence. The time frames and frequency of the TBSs were arranged with the study participants. Times for TBSs were Monday to Saturday from 9 am until 8 pm. If the study participant was not reached at the agreed time, up to four attempts at 1-week intervals were conducted. Those still unavailable following five attempts were considered to have an incomplete ETC (meaning the on-site counselling alone or on-site counselling in combination with one to three TBSs). ETC was conducted by two senior researchers (BN, EWG) who were not involved in the routine treatment of the ED patients. Both researchers were trained in tobacco control interventions by an independent research and teaching institute on the therapy of addictive disorders. A fidelity check on the adherence with recent guidelines was performed for each study participant and the fidelity checks were analysed within a 90-min supervision session by a therapist every 4 weeks.

### Follow-up

The primary study outcome was 7-day abstinence at the 12-month follow-up. Additionally the 7-day abstinence at 1, 3 and 6 months was evaluated independently of the study arm by staff members who were blinded to the group assignment. Study participants were additionally asked for changes in address, telephone number, and email address in order to minimise attrition due to inappropriate reachability. Telephone interviews were arranged at times that were convenient for study participants. Telephone interviews took place Monday to Friday from 10 am to 9 pm and on Saturdays from 10 am to 6 pm. At each time point, three attempts were made to reach participants by telephone before a brief questionnaire was posted. Those unreachable after these four attempts were categorised, at the specific time point, as “discontinued follow-up”, but were contacted at the next time point with the same procedure. Follow-up telephone interviews were carried out between November 2005 and January 2008. To validate the self-reported smoking intensity at the 12-month follow-up, exhaled carbon monoxide concentration (CO) was measured in a subsample of 100 study participants living in the greater Berlin area. Between March and November 2007 CO measures took place in participants’ homes or in the ED if study participants were willing to again visit the ED. One staff member contacted 188 study participants before this number could be reached (28 declined to participate, in 34 no appointment for a CO measurement within 1 month was possible and 26 could not be contacted or did not attend the scheduled appointment). CO was measured using a NeoMed EC50 CO analyser (Smokerlyser series no. 41693; NeoMed Medical Technology, Korschenbroich, Germany). The cut-off of was chosen as 6.5 parts per million (ppm) CO in the exhaled air as previous reported.^26^

### Statistical analysis

All binary and categorical variables are shown as frequencies. Metric variables are shown as medians (ranges). In study participants with complete baseline screening, smoking-related variables and socioeconomic parameters were compared across the ETC and control groups using the Mann–Whitney U test for metric variables and the χ^2^ test for nominal respectively ordinal variables. The Jonckheere–Terpstra test was used to compare metric variables and the Mantel–Haenszel test for trend was used to compare nominal respectively ordinal variables across the three stages of motivation to change behaviour. Intention-to-treat analysis of the primary study outcome was by binary logistic regression analysis, adjusted for age and gender (proc logistic in SAS V. 9.1 (SAS Institute, Cary, North Carolina, USA) with the contrast-statement to evaluate the effect of ETC stratified for motivation). Study participants not reached at follow-up were assumed to be continuous smokers. Subgroup analysis in the 1012 study participants with complete baseline screening regarding 7-day abstinence at 12 months additionally included the degree of nicotine dependency, the latter mentioned as independent predictor of smoking cessation.^27^ ^28^ A p⩽0.05 (two-sided) was determined as being significant. All statistical analysis was performed using SAS software.

## RESULTS

**Figure 2:**
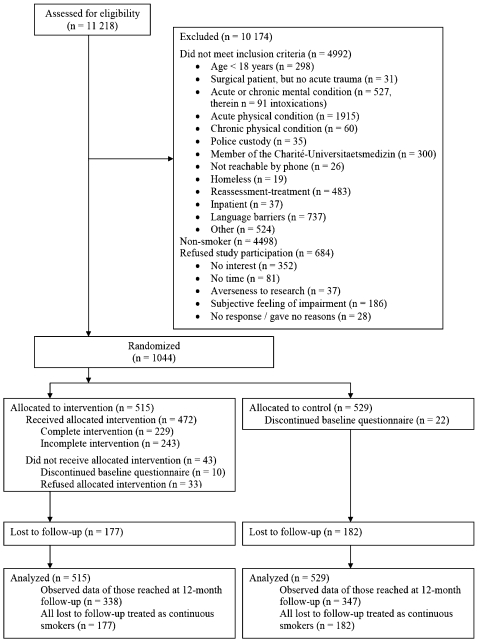
Flowchart of all patients, baseline enrolment and follow-up.

In total, 11 218 consecutive patients were assessed for eligibility (fig 2). Overall, 4992 (44.5%) did not meet the inclusion criteria. Of 6226 patients questioned about their smoking status, 4498 (72.2%) were non-smokers. This left 1728 potential study participants. Of these, 684 (39.6% of 1728) refused study participation. Reasons for non-participation are given in fig 2. Overall, 1044 patients were randomised (515 in the ETC and 529 in the control group). There was no difference between the ETC group and the control group with regard to the proportion of male study participants (311/515, 60.4% vs 319/529, 60.3%, p = 0.98), motivation to change smoking behaviour (286/515, 55.5% unmotivated, 165/515, 32.0% ambivalent and 64/515, 12.4% (percentage does not sum up to 100% because of rounding error) motivated smokers vs 294/529, 55.6% unmotivated, 166/529, 31.4% ambivalent and 69/529, 13.0% motivated smokers, Mantel–Haenszel test for trend: p = 0.90) and the median age at baseline (29 (range 18–78) years vs 30 (range 18–72) years, p = 0.35).

In the ETC group 10 study participants, and in the control group 22 study participants, discontinued baseline screening after the randomisation, leaving 1012 study participants with complete baseline screening. Another 33 patients in the ETC group (6.4% of 515) refused on-site counselling or immediately left the study site. Table 1 shows baseline patient characteristics in the ETC group compared to the control group in those 1012 study participants with complete baseline screening. Significant differences were found in the median number of cigarettes smoked per day (ETC group 15 (range 1–60) compared to 16 (range 1–50) in the control group, p = 0.04) and regarding size of household (p = 0.03). The majority of study participants (51.4%) had smoked for more than 10 years, and more than half had started smoking before age 17.

**Table 1:** Basic patients’ characteristics and comparison between Emergency Department-initiated tobacco control (ETC) group and control group in patients with complete baseline screening, n = 1012

Parameter	All patients	ETC group	Control group	p Value
	n = 1012 (100%)	n = 505 (49.9%)	n = 507 (50.1%)	
Age in years, median (range)	30 (18–78)	29 (18–78)	30 (18–72)	0.35
Gender, n (%):				
Male	614 (60.7)	308 (61.0)	306 (60.4)	0.84
Female	398 (39.3)	197 (39.0)	201 (39.6)	
Motivation to change smoking behaviour, n (%)*:				
Unmotivated smokers	557 (55.0)	280 (55.4)	277 (54.6)	
Ambivalent smokers	327 (32.3)	163 (32.3)	164 (32.3)	0.73†
Motivated smokers	128 (12.6)‡	62 (12.3)	66 (13.0)‡	
No. of cigarettes smoked per day during the last 7 days, median (range)	15 (1–60)	15 (1–60)	16 (1–50)	**0.04**
Nicotine dependency, n (%)§:				
Low	424 (41.9)	222 (44.0)	202 (39.8)	
Medium	241 (23.8)	112 (22.2)	129 (25.4)	0.36†
High	347 (34.3)	171 (33.9)‡	176 (34.7)‡	
Smoking duration, n (%):				
<1 year	25 (2.5)	16 (3.2)	9 (1.8)	
1–3 years	81 (8.0)	41 (8.1)	40 (7.9)	0.39†
4–10 years	386 (38.1)	191 (37.8)	195 (38.5)	
>10 years	520 (51.4)	257 (50.9)	263 (51.9)‡	
Age of smoking onset, n (%):				
<14 years	151 (14.9)	80 (15.8)	71 (14.0)	
14–16 years	375 (37.1)	192 (38.0)	183 (36.1)	
17–18 years	227 (22.4)	105 (20.8)	122 (24.1)	0.36†
19–30 years	245 (24.2)	121 (24.0)	124 (24.5)	
>30 years	14 (1.4)	7 (1.4)	7 (1.4)‡	
Attempts to quit smoking during the last 12 months, n (%):				
None	584 (57.7)	275 (54.5)	309 (60.9)	
1	232 (22.9)	126 (25.0)	106 (20.9)	0.08†
2–5	159 (15.7)	84 (16.6)	75 (14.8)	
>5	37 (3.7)	20 (4.0)§	17 (3.4)	
Partner smoking, n (%):				
No	280 (27.7)	134 (26.5)	146 (28.8)	
Yes	434 (42.9)	217 (43.0)	217 (42.8)	0.66
No partnership	298 (29.4)	154 (30.5)	144 (28.4)	
Hazardous alcohol consumption, n (%)¶:				
Yes	349 (34.5)	177 (35.0)	172 (33.9)	0.71
No	663 (65.5)	328 (65.0)	335 (66.1)	
Illicit drug use (last 12 months), n (%):				
None	614 (60.7)	299 (59.2)	315 (62.1)	
1–3 times	158 (15.6)	83 (16.4)	75 (14.8)	0.58†
4 times up to weekly	131 (12.9)	70 (13.9)	61 (12.0)	
Several times per week to daily	109 (10.8)	53 (10.5)	56 (11.0)‡	
Types of drugs in illicit drug users (n = 398), n (%):				
Cannabis only	201 (50.5)	104 (50.5)	97 (50.5)	
Ecstasy/designer drugs only	15 (3.8)	4 (1.9)	11 (5.7)	0.18
Cocaine only	1 (0.3)	1 (0.5)	0 (0.0)	
All other combinations	181 (45.5)‡	97 (47.1)	84 (43.8)	
School education, n (%):				
Discontinued	13 (1.3)	10 (2.0)	3 (0.6)	
10 years	484 (47.8)	232 (45.9)	252 (49.7)	0.18
11–13 years	501 (49.5)	256 (50.7)	245 (48.3)	
In school education	14 (1.4)	7 (1.4)	7 (1.4)	
Current occupation, n (%):				
Full time working	495 (48.9)	252 (49.9)	243 (47.9)	
Unemployed	96 (9.5)	47 (9.3)	49 (9.7)	0.82
All other	421 (41.6)	206 (40.8)	215 (42.4)	
Marital status, n (%):				
Married, living with the partner	153 (15.1)	68 (13.5)	85 (16.8)	
Married, living separate	34 (3.4)	19 (3.8)	15 (3.0)	0.42
Widowed/divorced	82 (8.1)	39 (7.7)	43 (8.5)	
Single	743 (73.4)	379 (75.0)	364 (71.8)‡	
Size of household, n (%):				
1 person	388 (38.3)	210 (41.6)	178 (35.1)	**0.03**
>1 person	624 (61.7)	295 (58.4)	329 (64.9)	
Net family income/month, n (%):				
Below average**	473 (46.7)	239 (47.3)	234 (46.2)	0.54
Above average**	298 (29.4)	141 (27.9	157 (31.0)	
No data	241 (23.8)‡	125 (24.8)	116 (22.9)‡	
Family doctor, n (%):				
Yes	724 (71.5)	359 (71.1)	365 (72.0)	0.75
No	288 (28.5)	146 (28.9)	142 (28.0)	
Visits to the family doctor (last 12 months) in patients with a family doctor (n = 724), n (%):				
None	63 (8.7)	28 (7.8)	35 (9.6)	
1 or 2	353 (48.8)	173 (48.2)	180 (49.3)	0.32†
3 or more	308 (42.5)	158 (44.0)	150 (41.1)	
Medical status, n (%):				
Surgical	485 (47.9)	238 (47.1)	247 (48.7)	0.61
Internal	527 (52.1)	267 (52.9)	260 (51.3)	

Significant values are in bold.

*“When do you wish to stop smoking?” (“not within the next 6 months” = unmotivated smokers, “within the next 6 months but not within the next 4 weeks” = ambivalent smokers and “within the next 4 weeks” = motivated smokers); †Mantel–Haenszel test for trend; ‡does not sum up to 100% because of rounding error; §measured with the Fagerstroem test for nicotine dependency; “low” = 0–2 points, “medium” = 3–4 points, “high” = 5–10 points; ¶measured with the AUDIT-PC questionnaire; “no” = 0 to 4 points, “yes” = 5 to 20 points; **average = mean net household income per month in Berlin in 2004 (ie, €1725).^29^

AUDIT-PC, Alcohol Use Disorder Identification Test – Primary Care.

Differences between motivation to change smoking groups (see table 2) in those 1012 study participants with complete baseline screenings were found regarding time variables (longer smoking duration and earlier onset of smoking in less motivated smokers) and attempts to quit smoking (more attempts in higher motivated smokers).

**Table 2:** Age, gender and smoking related variables stratified according to the motivation to change behaviour in patients with complete baseline screening, n = 1012

Parameter	Stratified according to the motivation to change smoking behaviour at baseline*			p Value
	Unmotivated smokers	Ambivalent smokers	Motivated smokers	
	n = 557 (55.0%)	n = 327 (32.3%)	n = 128 (12.6%)†	
Age in years, median (range)	29 (18–72)	30 (18–73)	30.5 (19–78)	0.48‡
Gender, n (%):				
Male	339 (60.9)	192 (58.7)	83 (64.8)	0.70§
Female	218 (39.1)	135 (41.3)	45 (35.2)	
No. of cigarettes smoked per day during the last 7 days, median (range)	15 (1–60)	16 (1–50)	10 (1–60)	0.12‡
Nicotine dependency, n (%)¶:				
Low	229 (41.1)	138 (42.2)	57 (44.5)	0.49§
Medium	133 (23.9)	79 (24.2)	29 (22.7)	
High	195 (35.0)	110 (33.6)	42 (32.8)	
Smoking duration, n (%):				
<1 year	6 (1.1)	8 (2.4)	11 (8.6)	**0.003**§
1–3 years	45 (8.1)	23 (7.0)	13 (10.2)	
4–10 years	212 (38.1)	128 (39.1)	46 (35.9)	
>10 years	294 (52.8)†	168 (51.4)†	58 (45.3)	
Age of smoking onset, n (%):				
<14 years	83 (14.9)	46 (14.1)	22 (17.2)	**0.043**§
14–16 years	224 (40.2)	112 (34.3)	39 (30.5)	
17–18 years	122 (21.9)	78 (23.9)	27 (21.1)	
19–30 years	123 (22.1)	87 (26.6)	35 (27.3)	
>30 years	5 (0.9)	4 (1.2)†	5 (3.9)	
Attempts to quit smoking during the last 12 months, n (%):				
None	411 (73.8)	143 (43.7)	30 (23.4)	**<0.001**§
1	89 (16.0)	97 (29.7)	46 (35.9)	
2–5	44 (7.9)	80 (24.5)	35 (27.3)	
>5	13 (2.3)	7 (2.1)	17 (13.3)†	
Partner smoking, n (%):				
No	134 (24.1)	99 (30.3)	47 (36.7)	0.083
Yes	261 (46.9)	130 (39.8)	43 (33.6)	
No partnership	162 (29.1)†	98 (30.0)†	38 (29.7)	

Significant values are in bold.

*“When do you wish to stop smoking?” (“not within the next 6 months” = unmotivated smokers, “within the next 6 months but not within the next 4 weeks” = ambivalent smokers and “within the next 4 weeks” = motivated smokers); †does not sum up to 100% because of rounding error; ‡Jonckheere–Terpstra test; §Mantel–Haenszel test for trend; ¶measured with the Fagerstroem test for nicotine dependency; “low” = 0–2 points, “medium” = 3–4 points, “high” = 5–10 points.

For study participants in the ETC group, table 3 shows the characteristics of the ETC as well as differences in subgroups stratified for motivation to change smoking behaviour. The overall median time of ETC for the 472 study participants who received at least the on-site counselling was 30 (range 1–99) min, with the on-site counselling taking a median duration of 13 (range 1–45) min. Less than half (230 out of 515, 44.7%) of the ETC group completed the ETC. Approximately 15% of the 472 ETC participants who received at least the on-site counselling chose nicotine replacement therapy. Significant differences in different subgroups of motivation to change smoking behaviour were found regarding the time of the on-site counselling and the overall duration of ETC, the proportion of study participants who received nicotine replacement therapy (NRT) or agreed on a quitting day. Motivated smokers had the longest ETC times and more motivated smokers, compared to ambivalent or unmotivated smokers, agreed on a quitting day and/or received NRT.

**Table 3:** Emergency Department-initiated tobacco control (ETC) in study participants with complete baseline screening, allocated to intervention and stratified for the motivation to change smoking behaviour, n = 515

	All patients in the ETC group	Patients in the ETC group stratified according to the motivation to change smoking behaviour at baseline*			p Value
		Unmotivated smokers	Ambivalent smokers	Motivated smokers	
	n = 515 (100%)	n = 286 (55.5%)	n = 165 (32.0%)	n = 64 (12.4%)†	
No. of contacts during ETC, n (%):					
0 (no ETC)	43 (8.3)	24 (8.4)	16 (9.7)	3 (4.7)	
1 (“only” on-site counseling)	64 (12.4)	36 (12.6)	19 (11.5)	9 (14.1)	
2	48 (9.3)	30 (10.5)	11 (6.7)	7 (10.9)	0.31‡
3	61 (11.8)	41 (14.3)	15 (9.1)	5 (7.8)	
4	69 (13.4)	32 (11.2)	27 (16.4)	10 (15.6)	
5 (complete ETC)	230 (44.7)†	123 (43.0)	77 (46.7)†	30 (46.9)	
Time of the on-site counselling in min (n = 472)§, median (range)	13 (1–45)	12 (1–45)	15 (1–35)	17 (1–35)	**0.002**¶
Only on-site counselling of 1–3 min (n = 472), n (%):§					
Yes	20 (4.2)	11 (4.2)	6 (4.0)	3 (4.9)	0.87‡
Overall time of ETC in min (n = 472), median (range)§	30 (1–99)	27 (1–75)	34 (2–99)	35 (1–77)	**<0.001**¶
Received nicotine replacement therapy (n = 472), n (%):§					
Yes	70 (14.8)	28 (10.7)	28 (18.8)	14 (23.0)	**0.004**‡
Agreed on a quitting day during the on-site counselling (n = 472), n (%):§					
Yes	83 (17.6)	39 (14.9)	28 (18.8)	16 (26.2)	**0.036**‡

Significant values are in bold.

*“When do you wish to stop smoking?” (“not within the next 6 months” = unmotivated smokers, “within the next 6 months but not within the next 4 weeks” = ambivalent smokers and “within the next 4 weeks” = motivated smokers); †does not sum up to 100% because of rounding error; ‡Mantel–Haenszel test for trend; §only those study participants who were allocated to intervention and received at least the on-site counselling; ¶Jonckheere–Terpstra test.

At the 12-month follow-up 685 participants (65.6% of 1044) could be reached. As shown in fig 2, 177 out of 515 (34.4%) study participants in the ETC group were lost to follow-up (therein 10 study participants who discontinued the baseline questionnaire) and 182 out of 529 study participants (34.4%) in the control group were lost to follow-up (therein 22 study participants who discontinued the baseline questionnaire). Table 4 shows the results of the non-responder analysis. Responders were a median age of 1 year older, more often women, less nicotine dependent, less often characterised by additional substance use, more highly educated and were more likely to have a family doctor.

**Table 4:** Comparison between responder and non-responder groups at 12 months, n = 1044

Parameter	Responders	Non-responders	p Value
	n = 685 (65.6%)	n = 359 (34.4%)	
Randomisation, n (%):			
ETC group	338 (49.3)	177 (49.3)	0.990
Control group	347 (50.7)	182 (50.7)	
Age in years, median (range)	30 (18–81)	29 (18–78)	**<0.001**
Gender, n (%):			
Male	396 (57.8)	234 (65.2)	**0.021**
Female	289 (42.2)	125 (34.8)	
No. of cigarettes smoked per day during the last 7 days (n = 1012), median (range)	15 (1–60)	15 (1–60)	0.124
Nicotine dependency (n = 1012), n (%):*			
Low	310 (45.3)	114 (34.9)	
Medium	158 (23.1)	83 (25.4)	**0.002†**
High	217 (31.7)‡	130 (39.8)‡	
Motivation to change smoking behaviour, n (%)§:			
Unmotivated smokers	375 (54.7)	205 (57.1)	
Ambivalent smokers	227 (33.1)	104 (29.0)	0.905†
Motivated smokers	83 (12.1)‡	50 (13.9)	
Attempts to quit smoking during the last 12 months (n = 1012), n (%):			
None	395 (57.7)	189 (57.8)	
1	153 (22.3)	79 (24.2)	0.667†
2–5	111 (16.2)	48 (14.7)	
>5	26 (3.8)	11 (3.4)‡	
Hazardous alcohol consumption (n = 1012), n (%):¶			
Yes	221 (32.3)	128 (39.1)	**0.031**
No	464 (67.7)	199 (60.9)	
Illicit drug use (last 12 months) (n = 1012), n (%):			
None	430 (62.8)	184 (56.3)	
1–3 times	109 (15.9)	49 (15.0)	**0.006†**
4 times up to weekly	84 (12.3)	47 (14.4)	
Several times per week to daily	62 (9.1)‡	47 (14.4)‡	
School education (n = 1012), n (%):			
Discontinued	5 (0.7)	8 (2.4)	
10 years	311 (45.4)	173 (52.9)	**<0.001**
11–13 years	364 (53.1)	137 (41.9)	
In school education	5 (0.7)‡	9 (2.8)	
Family doctor (n = 1012), n (%):			
Yes	507 (74.0)	217 (66.4)	**0.012**
No	178 (26.0)	110 (33.6)	

*Measured with the Fagerstroem test for nicotine dependency, “low” = 0–2 points, “medium” = 3–4 points, “high” = 5–10 points; †Mantel–Haenszel test for trend; ‡does not sum up to 100% because of rounding error; §“When do you wish to stop smoking?” (“not within the next 6 months” = unmotivated smokers, “within the next 6 months but not within the next 4 weeks” = ambivalent smokers and “within the next 4 weeks” = motivated smokers); ¶measured with the AUDIT-PC questionnaire: “no” = 0 to 4 points, “yes” = 5 to 20 points.

AUDIT-PC, Alcohol Use Disorder Identification Test – Primary Care; ETC, Emergency Department-initiated tobacco control.

**Figure 3:**
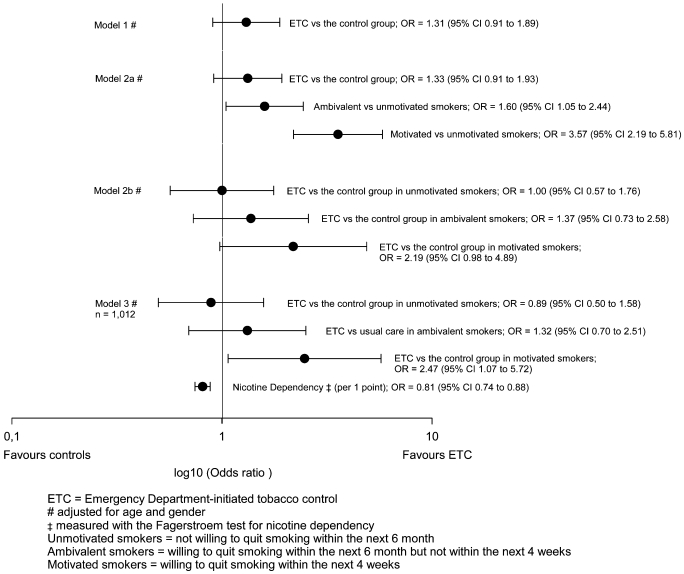
ORs and their corresponding 95% CIs of the effect of Emergency Department-initiated tobacco control vs the control group on 7-day abstinence at 12-month follow-up adjusted for age and gender, n = 1044 (models 1 and 2) and subgroup analysis in patients with complete baseline screening, n = 1012 (model 3).

The results on 7-day abstinence in the ETC group versus the control group based on intention-to-treat were overall 73/515 (14.2%) vs 60/529 (11.3%). In unmotivated smokers 26/286 (9.1%) in the ETC group and 27/294 (9.2%) in the control group were abstinent, whereas in ambivalent smokers 26/165 (15.8%) in the ETC group and 20/166 (12.0%) in the control were abstinent. In motivated smokers 21/64 (32.8%) in the ETC group and 13/69 (18.8%) in the control group were abstinent, respectively. This translates, after adjustment for age and gender, to an overall odds ratio of 1.31 (95% CI 0.91 to 1.89), p = 0.15 for the ETC group vs the control group (model 1 in fig 3). Additional adjustment for motivation to change smoking behaviour did not change the estimate of ETC vs the control group. The estimate for motivation to change smoking behaviour was highly significant (p for trend <0.001, model 2a in fig 3). The interaction between ETC and motivation (model 2b in fig 3) revealed no effect of ETC vs the control group in unmotivated smokers, a non-significant positive effect of ETC vs the control group in ambivalent smokers and a more than twofold non-significant effect of ETC vs the control group in motivated smokers. The test for trend for the interaction of ETC and motivation was not significant, p = 0.29.

A subgroup analysis in 1012 study participants with complete baseline screening (model 3 in fig 3) revealed an independent negative effect of nicotine dependence on 7-day abstinence at 12 months while the effect of ETC vs the control group in motivated smokers became significant. The interaction of ETC and motivation remained non-significant, p for trend = 0.14, respectively.

Of the 100 study participants with CO measured in the exhaled air at 12-month follow-up, 1 dataset could not be used because of technical failure of the CO measurement. Of the remaining 99 study participants 18 reported 7-day abstinence (see table 5). Sensitivity was 80.2% and specificity was 88.9%. The area under the receiver-operated curve (ROC) equalled 0.880 (95% CI 0.776 to 0.984).

**Table 5:** Carbon monoxide in parts per million (ppm) in the exhaled air at 12-month follow-up, n = 99

CO concentration*	Self-reported smokers	Self-reported non-smokers†	n (%)
⩾7 ppm	65 (80.2%)	2 (11.1%)	67 (67.7%)
0–6.5 ppm	16 (19.8%)	16 (88.9%)	32 (32.3%)
n (%)	81 (100%)	18 (100%)	99 (100%)

*Measured with a NeoMed EC50 CO analyser (see Methods); †7-day abstinence.

## DISCUSSION

These results confirm previous findings of no overall effect of ETC.^5^ ^6^ ^8^^–^10 Patients’ motivation to quit was a strong predictor of smoking cessation at follow-up as previously reported.^11^ Concerning unmotivated smokers, our approach does not seem to have any advantages over usual care. Thus, one consequence of our findings might be to limit ETC in unmotivated smokers to brief advice. Law and Tang^30^ in their analysis of 188 trials found a smoking cessation rate of 2% (95% CI 1% to 3%) following personal advice and encouragement to stop smoking given by doctors during a single routine consultation. Silagy,^24^ in a meta-analysis of 16 trials of brief advice versus no advice (or usual care), revealed a small but significant increase in the cessation rate of about 2.5%. Taking the lower limit of the 95% CI of a single routine consultation,^30^ these findings would translate into a number needed to treat (NNT) of 100, or an overall consultation time of 100–300 min for 1 additional smoking cessation.^2^ During on-site counselling in this investigation, several unmotivated smokers suggested intervals for the telephone booster follow-up sessions of 6 to 12 months (which could not be scheduled because of the study protocol). We interpreted this suggestion as a request for a long-term, low threshold tobacco control program. Therefore, another option might be to evaluate, in future studies, whether unmotivated smokers treated in EDs profit from a longer-term supportive therapeutic regimen, initiated on-site and maintained thereafter (eg, by a general practitioner; around 72% of unmotivated smokers had a family doctor, of these 90% with a minimum one visit during the last 12 months; data not shown).

In ambivalent smokers, our results showed a non-significant benefit of tobacco control over standard care with a 3.8% difference in 7-day abstinence rates at 12 months in an intention-to-treat analysis. The overall study was not designed to demonstrate differences in subgroups. Assuming clinical but not statistical significance due to an inappropriate number of study participants in this subgroup, the results would translate into a NNT of around 26 and a median treatment time of 884 min (the median time for 1 ETC in ambivalent smokers in this investigation was 34 min) for 1 additional smoking cessation. Thus, for ambivalent smokers who account for one third of the ED patients, alternative treatment regimes must be developed.

In motivated smokers we observed a 14.0% difference in 7-day abstinence rates at 12 months, with a NNT of around 7. This difference is based on a relatively small number of observations and should be interpreted cautiously. However, it would translate into an overall median treatment time of 245 min (the median time for one ETC in motivated smokers in this investigation was 35 min) to achieve one additional smoking cessation at 12 months. This finding would be consistent with the overall time for one additional smoking cessation after brief advice in unmotivated smokers (see above). In this investigation, being more highly motivated was associated with a shorter smoking duration and a higher age of smoking onset. Higher motivated smokers already had the most experience with quitting attempts within the last 12 months. When offered ETC, motivated smokers more intensively used the proposed consulting and were more often than unmotivated or ambivalent smokers willing to agree on a quitting day or to use NRT.

### Study limitations

Although study participants were randomised, stratified for age, gender and motivation to change behaviour, a statistical significant difference in the number of cigarettes per day between both study arms was observed. The median difference of one cigarette per day between groups, however, is probably of little clinical significance.

Because of the limited evidence on ETC at the time of study enrolment the sample size calculation was based on findings of inpatients showing an overall Peto odds ratio (OR) of 1.82 (95% CI 1.49 to 2.22) in tobacco control interventions with supportive contacts for at least 1 month compared with control condition.^13^ An update of this meta-analysis in 2007 based on 17 trials found a weaker overall effect of tobacco control (Peto OR = 1.65 (95% CI 1.4 to 1.9)).^31^ Thus, the study size calculation for this investigation was probably based on overestimated findings and led to incorrect sample sizes. ETC may be less effective than tobacco control in non-ED patients, due to a high prevalence of conjoint substance use and a cluster of further medical and social risks in ED patients. Another reason for the overall failure of ETC may be its initial application in an often hectic setting, one that hinders the establishment of a therapeutic relationship that is significant enough to assist the patient through the tobacco quitting process.

Findings from this study regarding baseline as well as follow-up enrolment must be seen as being impacted by potential selection bias during study participation assessment, incomplete allocation and incomplete ETC interventions. Richman *et al* reported complete allocation to intervention in their ED-based RCT in 152 out of 216 eligible patients (70.4%) and a 3-month follow-up of 103 (67.8%) study participants.^5^ Referral to a smoking cessation programme was accepted by less than half of 1095 smokers in the ED-based feasibility study by Cummings *et al*.^7^ Schiebel and Ebbert found in their feasibility study that 152 out of 212 smokers who initially indicated interest in quitting decided not to participate in their study and of those 39 finally randomised, 19 (48.7%) could not be reached or refused the 6-month follow-up.^8^ In our investigation, selection bias of those finally agreeing to study participation (1044 out of 1728) may have led to a positive sampling of those being more susceptible to health promotion since more than half of those smokers who refused participation were not interested in a study on tobacco control. Recall biases during follow-up may have further impacted on the validity of the results. But we assume that selection biases through discontinuation of the follow-up—in this study strongly associated with the degree of nicotine dependency as well as additional substance use parameters but independent of the motivation for behavioural change—was adequately controlled through our statistical approach.

The validation of self-reported smoking intensity through CO measurement in the exhaled air was conducted in a subsample of 100 out of 188 study participants leaving 99 datasets for analysis. Although validity in the subsample was satisfactory, the overall validity of the self-reported results on smoking cessation still remains questionable. But, our finding on the proportion of smoking deceivers (self-reported non-smokers chemically classified as smokers) was in the range of 4.0% and 13.0% smoking deceivers evaluated in previous studies on the validation of self-reported smoking.^30^ ^32^ ^33^

**What this paper adds**

- Although there is evidence that the smoking prevalence of emergency department (ED) patients exceeds the smoking prevalence in the population and a joint statement of US Emergency Medicine Organizations encourages administrators to implement tobacco control services, the effectiveness of such services is still unclear.
- In a randomised controlled trial in more than 1000 ED patients with a median smoking intensity of 15 cigarettes per day, ED-initiated tobacco control (ETC) showed a non-significant overall effect on 7-day abstinence at 12 months.
- Unmotivated smokers do not seem to profit from ETC while, in ambivalent and motivated smokers, a non-significant clinical effect of ETC was observed.

Despite these limitations, which might reflect the reality of future implementation into clinical routine, our study clearly showed the feasibility of ETC during routine activity in an urban ED. ETC, in the form tested in this investigation, appears to enhance the patient’s self-motivation in ambivalent and motivated smokers. The negative impact of nicotine dependence probably reflects the limiting (physical) factors in patient baseline characteristics. Systematically evaluating the benefit of nicotine replacement therapy in ETC services might adequately address these limiting factors in the future. Nicotine replacement therapy may further reduce attrition rates with ETC services since in this investigation higher nicotine dependency, but not lower motivation to change smoking behaviour, was associated with loss to follow-up.

## 

This study was approved by the Ethical Committee Board of Charité – Universitaetsmedizin Berlin, Berlin, Germany.
